# To organise or not to organise? Understanding search strategy preferences using Lego building blocks

**DOI:** 10.1177/17470218211040724

**Published:** 2021-09-02

**Authors:** Mona JH Zhu, Evan F Risko

**Affiliations:** Department of Psychology, University of Waterloo, Waterloo, Ontario, Canada

**Keywords:** Spatial organisation, spatial cognition, spatial decisions, embedded cognition

## Abstract

Humans routinely organise or reconfigure the environment as part of their everyday activities, such as placing a set of keys in a designated location to reduce the need to remember its location. This type of spatial organisation is widely thought to reduce both the physical and cognitive demands of a task to allow individuals to perform tasks more easily. Although spatial organisation can be a useful strategy when searching for items in the environment, individuals do not always choose to utilise these organisational strategies when carrying out everyday tasks. Across three experiments, we examined individuals’ preference for spatial organisation in the context of a real-world search task, and the degree to which individuals engaged in time- and effort-based cost–benefit analysis to inform whether to choose between an organisation-based or non-organisation-based search strategy. We found that individuals’ strategy preferences could be explained by the perceived task time associated with each strategy, but not perceived task effort. However, even statistically controlling for relative perceived task time or reported effort, participants showed a strong systematic preference against organisation prior to engaging in the task, and, post-task, a strong preference towards organisation. Implications for understanding individuals’ use of spatial organisation are discussed.

## Introduction

A portion of our everyday activities involves interacting with objects and making decisions about how to arrange them in space. *How* we arrange our environments can also exert influence on the way we behave and process information (Bernstein & Turban, 2018; Norman, 1988; Roster et al., 2016; Thaler & Sunstein, 2008). A growing body of research supports the idea that systematic organisation of objects in our environments can have a number of benefits for task performance (Kirsh, 1995, 1996; Solman & Kingstone, 2017a, 2017b). By observing how experts arrange objects in their workspace, Kirsh (1995) argued that spatial organisation can help to highlight or obscure certain choices in the environment, thereby reducing the amount of time or effort individuals spend on planning given actions. This allows individuals to reduce energetic costs associated with task performance by, e.g., placing relevant objects in more convenient locations. Indeed, there is evidence to suggest that when individuals are provided with opportunities to spontaneously arrange their environments, they often reconfigure their environments to make frequently encountered objects more accessible (Solman & Kingstone, 2017a), especially when doing so helps to reduce the amount of physical effort required to access these frequently used objects (Zhu & Risko, 2016). In addition to minimising energetic costs, spatial organisation can also help to reduce task-related cognitive demands. A commonly reported issue in disorganised spaces is that individuals have a harder time accessing relevant information or items (Malone, 1983; Williamson, 1998). Spatial organisation can mitigate this issue when target items are placed in strategic locations within a space which can, e.g., draw attention to these items (e.g., by placing important documents in an obvious location to serve as a reminder; Gilbert, 2015; Malone, 1983), facilitate more systematic search (e.g., placing cookware and utensils in the kitchen; Solman & Kingstone, 2017b), or allow for easier retrieval at a later time point (e.g., putting a set of keys in a designated location; Kirsh, 1995, 1996; Risko & Gilbert, 2016).

Although empirical work has demonstrated the utility afforded by spatial organisation in a number of situations, individuals do not always opt to organise their environments, even when doing so could result in objective energetic or cognitive savings. In a study conducted by Zhu and Risko (2016), participants completed a symbol copying task using two different pairs of writing utensils. Participants began each block by placing a single writing utensil in two designated locations on a desk—one close and one further away from the individual—in any order they preferred. To complete each trial within the block, participants would be given a visual cue that indicates which one of the writing utensils should be used; once participants copied the symbol, they completed the trial by putting the writing utensil back. After each block, participants switched to a different set of writing utensils, and could again spontaneously arrange them in any configuration in the designated locations. Critically, participants would be required to use some writing utensils in the sets far more frequently than others. By examining how individuals spontaneously placed the writing utensils at the start of each block, Zhu and Risko (2016) found that rather than moving more frequently used utensils closer to them, participants would often maintain the initial location of each writing utensil in space throughout the task, even when doing so required a further reach and resulted in slower task performance overall. In fact, participants only chose to configure their space more efficiently (i.e., moving frequently used writing utensils closer) when the physical cost associated with the reach increased substantially. This suggests that individuals may base their decisions about where to place objects in future scenarios on spatial decisions made in the past, rather than considering the objective energetic costs associated with these placements, if these costs are not sufficiently high.

This discrepancy between the potential benefits of spatial organisation and the lack of engagement in organisational activity was further demonstrated in a series of studies involving school-age children (Berry et al., 2019). Children were asked to perform a spatial working memory task that involved searching for a sequence of colour blocks among an array of blocks after the sequence was presented verbally. Notably, all children completed two conditions of the same task, one in which the array could be grouped such that blocks of the same colour category would be placed together in space, and the other wherein blocks were pseudo-randomly arranged so that adjacent blocks were always of different colours. Berry and colleagues (2019) found that children with a lower working memory capacity (WMC) performed better on the spatial working memory task when the blocks were organised by colour than when the blocks were pseudo-randomly placed. However, though children with a higher WMC performed better overall compared with children with a lower WMC, their performance did not differ across the two conditions. This suggests that children with a lower WMC benefitted more from systematically organised task environments than those with a higher WMC. However, when asked to rate the difficulty of the two conditions using Likert-type scales, the majority of children with higher WMC (85%) identified that the ordered condition was easier, whereas only 57% of the children with a lower WMC did so. That is, although children with a lower WMC benefitted more from an organised environment, they had more difficulty recognising the fact that spatial organisation facilitated their task performance. Equally counterintuitive was the fact that when presented with the opportunity to freely arrange their environment, children—regardless of their WMC—opted for a more random spatial arrangement than an organised one. In other words, although children with a higher WMC rated the organised condition as being easier, they did not choose to reorganise their task space in this manner. Altogether, these results suggest that individuals may not always choose to engage in spatial organisation despite being aware that doing so would be beneficial.

Although it may seem puzzling that individuals do not always choose to organise their task environments even though an organised environment would help facilitate task performance, it is important to note that while a well-structured space can help individuals to conserve effort when completing tasks, the construction and maintenance of that organised environment itself requires time and effort. As such, individuals’ decision to engage in organisation may reflect how they weigh the relative perceived costs and benefits of organisation against the perceived costs and benefits of operating in the resulting environment. From this perspective, we may expect individuals to engage in a kind of cost–benefit analysis, choosing to organise their environments when doing so is expected to result in a perceived net benefit. As such, a primary focus of the current research is to investigate the kinds of factors that may contribute to an individual’s decision to organise their space when provided with the opportunity to do so using a real-world search task.

## Experiment 1

We begin our investigation by examining potential factors that could influence individuals’ decision to use spatial organisation in a real-world search task involving Lego building blocks. Perceived task time may be an especially salient factor for individuals when considering whether to adopt a given task strategy. The notion that individuals opt for strategies that minimise time has received support across a number of studies within the strategy selection domain (Dunn & Risko, 2016; Gray & Boehm-Davis, 2000 ; Gray & Fu, 2004; Gray et al., 2006; Siegler & Lemaire, 1997). For example, according to the soft constraints hypothesis, individuals select strategies based primarily on a time-based cost–benefit analysis at least at relatively short time scales (Gray & Boehm-Davis, 2000; Gray & Fu, 2004; Gray et al., 2006). For instance, Gray and colleagues (2006) asked individuals to complete a memory matching task involving coloured blocks. Participants could either go back to access the original display or rely on their memory of the display for the matching task. Importantly, the cost (an increase in the amount of time required to access the original display) was manipulated. The authors found that when time costs were high, individuals shifted to a memory-intensive strategy (i.e., relying more heavily on their memory of the original display) in an effort to offset those time costs. While this research primarily focused on examining objective task completion time, perceived task time may be the more critical variable. For example, Dunn and Risko (2016) demonstrated, in the context of individuals selecting between reading a rotated display while remaining upright versus physically rotating their head, that individual’s selections appeared to more closely follow perceived time (and accuracy) than the objective time associated with each strategy.

Although time-minimisation could conceivably benefit individuals by, e.g., freeing up time for them to complete other goals or tasks, it is unlikely that time would be the only consideration when selecting a given strategy for a task; indeed, individuals can and do prioritise other factors over time in some cases (Dunn et al., 2019; Kool et al., 2010; Weis & Wiese, 2018). For example, Weis and Wiese (2019) demonstrated that an individual’s higher level goals will play a critical role when participants are choosing between alternative strategies. Specifically, the authors found that individuals’ use of a novel external tool that reduced internal processing in a cognitive task varied as a function of whether individuals were provided speed- or accuracy-based instructions. Given how strategy selection may be tied to task goals, it is worth noting that in the present investigation, task goals were completion-oriented (i.e., “complete the following task”) rather than explicitly performance-oriented (e.g., “go fast”), nor was performance incentivised (e.g., see Walsh & Anderson, 2011). Thus, individuals were left relatively unconstrained in how they were expected to perform the task in an effort to reveal their spontaneous strategy preferences. It is under these conditions that we think perceived task time would be a particularly salient consideration.

In Experiment 1, we asked participants to search for a subset of target pieces among a large pile of Lego building blocks. Before the task, participants were asked whether they would prefer to organise the pile before beginning the search task (e.g., categorising based on colour or by shape) or to proceed directly to completing the search task. After making this decision, we asked participants to provide time estimates for how long they expected to take to complete the entire task both with an organisation-based strategy (i.e., accounting for both the time it would take to organise the pieces and the time it would take to search for just the target ones) and without (i.e., the amount of time it would take to search for all target pieces from an unorganised pile). If individuals’ preferences were based on the perceived costs and benefits involved in the strategies available, then we would expect them to prefer an organisation-based strategy only when they expect it to take less time, and vice versa. During the task, participants were assigned to use either an organisation- or non-organisation-based search strategy, regardless of their stated preferences earlier. Importantly, in contrast with previous experiments that examined strategy selection where one strategy would be objectively more efficient (e.g., Zhu & Risko, 2016), we attempted to make the task such that participants would complete the task using roughly the same amount of time regardless of which strategy was used. In addition to examining individuals’ preference pre-task and measuring performance during the task, we also asked participants a number of follow-up questions in an attempt to gauge their experience during task performance, and whether experience with the task altered the perceived utility of each strategy.

### Methods

#### Participants

We recruited a total of 53 students (37 female) from the University of Waterloo to participate in our study for course credit. This sample size was determined based on ensuring that a minimum of 20 participants would be assigned to use each search strategy to determine whether task completion time was equal across strategies. The mean age of our sample was 20.83 years (*SD* = 1.31); all participants reported normal or corrected-to-normal vision. All participants in the reported studies provided informed consent prior to the study and were debriefed about the purpose of the studies upon completion. Ethical approval was obtained from the University of Waterloo Research Ethics Board.

Prior to exclusion, 28 participants were assigned the search-only strategy, while 25 were assigned the organisation-based strategy. After the exclusion criteria were applied, the number of participants was 21 and 20 in the respective conditions.

#### Materials and procedure

**Figure 1.**
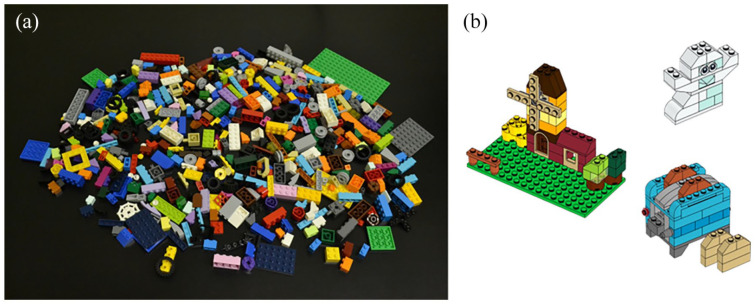
Photos of the total Lego set that participants must work with (a) as well as schematics of the structures that the target pieces will help to construct (b).

At the start of the study, participants were told that they would be completing a search task that involved searching for a subset of target pieces among a pile of Lego building blocks. They were then presented with the pile in front of them (see Figure 1a), which had a total of 483 pieces, as well as the instruction cards with the relevant pieces that they would be searching for (see https://www.lego.com/en-ca/themes/classic/building-instructions/10696 for examples of the instruction cards), consisting of a total of 161 target pieces. These target pieces were pieces made up of structures as shown in Figure 1b. Importantly, participants were strictly told that they were only to search for the relevant pieces, and that they must not construct the Lego structures as presented on the instruction cards.

After an overview of the task was provided, participants were told that they will be using one of the two strategies to search for the target pieces—to (a) directly search for the target pieces without any spatial organisation or (b) to organise the pile into categories based on some form of external feature (e.g., colour or shape) and then search for the target pieces—and they were asked to provide which of the two strategies they preferred. They were then asked to estimate how long it would take them to complete the task for each of the two strategies. For the organisation-based strategy, the estimated task time was broken into the amount of time they expected to spend on organisation only and the amount of time they expected to search for the pieces only; for the search-only strategy, only the total search perceived task time was provided.

After participants provided their strategy preference and respective time estimates, they were randomly assigned to complete the search strategy using one of the two strategies presented to them earlier. At this point, the experimenter provided more detailed instructions for completing the search task. Those assigned to the organisation-based strategy were told that they must complete the task in sequence such that they must organise all the pieces into colour categories before moving on to the search task. Note that individuals were only provided with the criterion of forming colour categories and were free to form as many or as few colour categories as they wished. However, those assigned to complete the search using the search-only strategy were told they must not form any systematic categories while they search for the pieces. The experimenter left the room while participants completed the search task; to measure objective task time and to ensure compliance, a GoPro HERO3 was mounted onto the wall to provide a bird’s eye perspective of the task space and was used to record participants’ performance throughout the study.

Once participants completed the search task, they were asked to provide a time estimate for how long it took them to complete the task using the assigned strategy, as well as how long they think it would take them using the alternate strategy that they were not assigned, similar to that in the pre-task. The estimate for the organisation-based strategy was again broken down into the amount of time they expect to spend on the organisation and search phase separately once a total estimate was provided. Following task time estimates, we also asked participants to describe the exact strategy they used when completing the search task to ensure that participants completed the task according to the instructions. We also asked them whether, given their current experience with the task, they would continue to use the same strategy they were assigned in the future as a measure of their updated strategy preference post-task. If participants answered no to this question, we asked them to elaborate and describe their preferred alternative strategy. Finally, participants were probed for suspicion regarding the purpose of the experiment before being debriefed.

### Results

Although we included data from all individuals for their pre-task responses, 12 individuals’ objective task time and post-task responses were removed from analyses due to equipment failure during the search task (7) or improper task completion (5). Unless noted otherwise, the pattern of results remained the same when these subjects were removed from post-task analyses.

All analyses were conducted using R (R Core Team, 2019). In addition to *p* values, we report Hedges’s *g* using the *effsize* package (Torchiano, 2019) as a measure of effect size for two-group comparisons wherever appropriate. Note that Hedges’s *g* is a correction applied to Cohen’s *d* that corrects for small sample sizes (Hedges, 1981); in other words, Hedges’s *g* and Cohen’s *d* are comparable measures of effect size. For all logistic regression models, we report odds ratios. In addition, 95% likelihood ratio confidence interval for each estimate in a logistic regression model was reported (Meeker & Escobar, 1995), as extracted using the *sjPlot* package (Lüdecke, 2018). The *sjPlot* package was also used to create and construct regression output tables. Aside from difference scores, all continuous variables are mean-centred. In addition, sum contrast coding was applied whenever categorical predictors were included in a model. When contrast coding is used in a given model, the model intercept represents the grand mean between the two conditions, rather than the mean of the reference condition.

We used the *lme4* package in R (Bates et al., 2015) to conduct mixed-effects model analyses when the same individuals made multiple responses (e.g., making multiple task time estimations across an experiment) using the default optimisers. We began with the simplest model (i.e., allowing for the intercept to vary per individual to account for within-in participant variation) and opted for more complex random effects structures (e.g., allowing both the intercept and slopes to vary per individual, as opposed to just the intercept) when there were sufficient observations in the data set for the model to converge, when it did not yield a singular model (an indication that the added slope explained negligible variance in the model), and when doing so improved the model fit (Bates et al., 2018; Matuschek et al., 2017). For linear mixed-effects models, maximum likelihood estimation was used in place of the default restricted maximum likelihood estimation. In cases where models failed to converge but the gradient was sufficiently small (i.e., <.002), we increased the number of iterations from the default 10,000 to 30,000 to reach convergence. As degrees of freedom for mixed-effects models can be difficult to estimate, *p* values are approximated using Wald *z* statistics, and 95% Wald confidence intervals are reported, as extracted using the *sjPlot* package (Lüdecke, 2018).

#### Objective task time

Individuals assigned to the search-only strategy spent 28.90 min (*SD* = 6.65) on average completing the search task, while those assigned to the organisation-based strategy spent 28.03 min (*SD* = 8.70). Note that the time reported for the organisation-based strategy includes both the time taken to organise all pieces in the workspace as well as to search for the target pieces. Task completion time was not statistically different between assigned task strategies, as indicated by Welch’s two-sample *t* test, *t*(35.57) = 0.36, *p* = .721, *g* = 0.11. On average, those assigned to the organisation-based strategy completed the organisation phase in 13.33 min (*SD* = 8.05).

#### Estimated task completion time

We compared individuals’ estimated task time for each strategy prior to the start of the task using a paired-sample *t* test. There was no significant difference between the overall task time estimates provided for search-only strategy (*M* = 28.07, *SD* = 13.80) versus the organisation-based strategy, *M* = 30.13, *SD* = 17.57, *t*(52) = 1.35, *p* = .184, *g* = 0.12.^1^ The mean estimated time for completing the organisation phase was 12.38 min (*SD* = 7.87).

As with the pre-task time estimates, there was also no significant difference between overall time estimates provided for the search-only (*M* = 33.98, *SD* = 11.53) and the organisation strategies (*M* = 31.49, *SD* = 13.31) when participants were asked to provide these estimates post-task, *t*(40) = 1.04, *p* = .303, *g* = 0.20. The organisation phase was estimated to take 15.22 min (*SD* = 9.65). Figure 1 provides a visual comparison of both the objective task completion time for each strategy, as well as individuals’ perceived task time for each strategy pre- and post-task.

**Figure 2.**
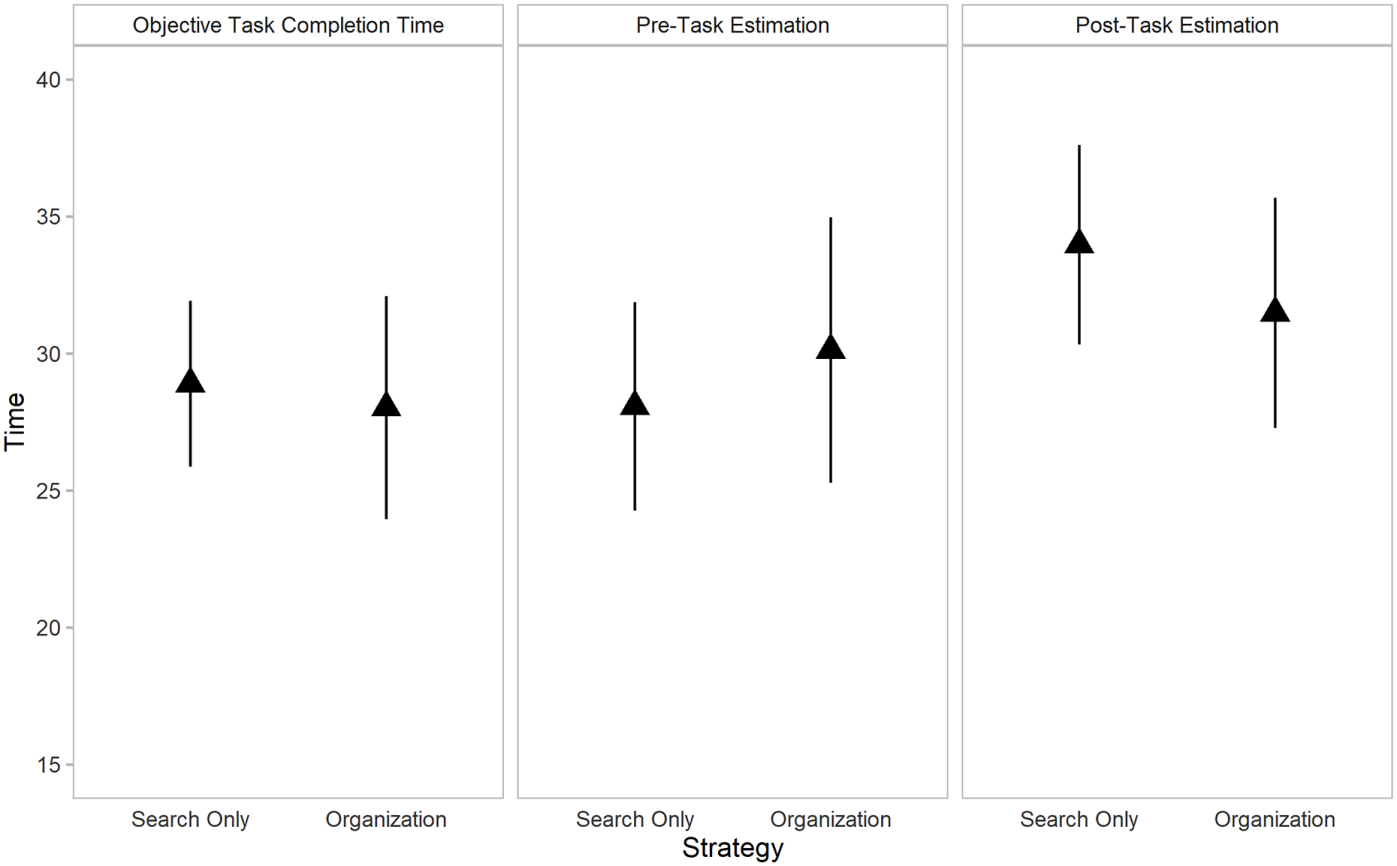
Objective task completion time as a function of assigned strategy (left) and estimated task time using either strategy (centre and right) in Experiment 1. Error bars represent 95% confidence intervals.

For descriptive purposes, of the 53 total participants, 20 perceived an organisation-based strategy to be faster, 25 perceived the search-only strategy to be faster, and the remaining 8 perceived the two tasks to take the same amount of time pre-task. Post-task, 27 out of 41 participants perceived the organisation-based strategy to be faster, 10 perceived the search-only strategy to be faster, and 4 perceived them to take the same amount of time. Results for objective as well as estimated task completion time are shown in Figure 2.

#### Strategy preference

##### Pre-task strategy preference

First, we examined whether individuals had any systematic preferences for a given strategy pre-task. Interestingly, the majority of individuals (73.6%) preferred the search-only strategy over organisation, which was statistically different from no preference (i.e., 50%), χ^2^(1, *N* = 53) = 11.79, *p* < .001.

Next, we examined whether participants’ chosen strategies were predicted by the set of time estimates they provided. As mentioned previously, though overall time estimates provided for each strategy do not differ overall, it is possible that *relative* perceived time difference across strategies provided by each individual may drive their strategy preferences. To do so, we took the difference between the time estimates provided between the two strategies (i.e., time estimate for the search-only strategy minus that of the organise-first strategy) and used this difference score in a logistic regression model to predict whether individuals were more likely to choose an organise-first (coded as 1) or a search-only strategy (coded as 0). As such, a positive difference score would indicate that the organise-first strategy was thought to have taken less time, and vice versa. Results from this model supported our prediction; the less time individuals rated the organise-first strategy to take relative to the search-only strategy, the more likely they were to pick the organise-first strategy, *b* = 1.12, 95% confidence interval (CI) = [1.04, 1.24], *z* = 2.69, *p* = .007. Specifically, individuals were 1.12 times more likely to prefer the organisation-based strategy for every 1 min that this strategy was perceived to be faster relative to the search-only strategy. However, the intercept remained significant in the model; when relative perceived time across tasks was at 0, individuals were 3.03 times more likely to prefer the search-only strategy relative to the organisation-based strategy, *b* = 0.33, 95% CI = [0.15, 0.64], *z* = 3.05, *p* = .002. In other words, even when there was no difference in estimated task time between each strategy, individuals still had a preference for the search-only strategy.

##### Post-task strategy preference

**Figure 3.**
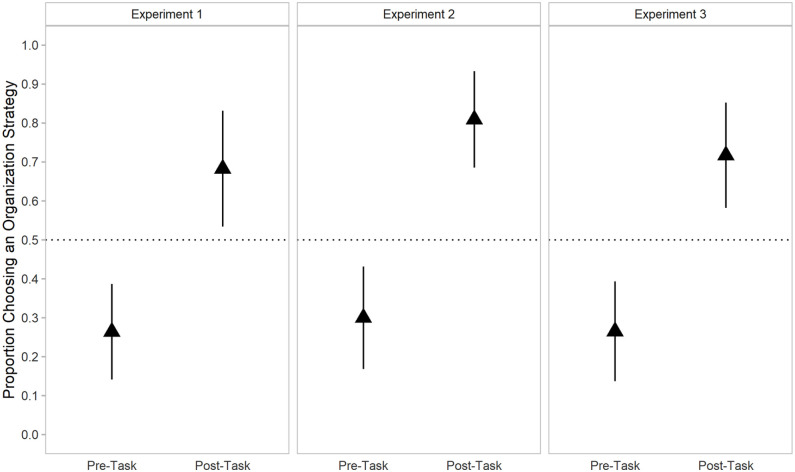
Mean proportion of individuals who preferred an organisation-based strategy pre-task and post-task in Experiment 1 through 3. Error bars represent 95% confidence intervals.

In addition to pre-task strategy preferences, we also examined participant’s strategy preference post-task. Participants were asked post-task whether they would use the same strategy as the one they were assigned pre-task, and if not, to describe the alternative strategy they would use, and their responses were coded. All participants provided either a search-only strategy or a strategy that incorporated organisation (e.g., organising pieces by shape instead of by colour). Surprisingly, whereas individuals preferred a search-only strategy pre-task, this preference was reversed post-task such that most individuals (68.3%) preferred an organisation-based strategy overall, χ^2^(1, *N* = 41) = 5.49, *p* = .019. Figure 3 provides a visual depiction of individuals’ pre- and post-task strategy preference.

Using the difference score between the time estimates provided post-task, a logistic regression revealed that time estimates significantly predicted post-task strategy preference. As with pre-task time estimates, there was a positive relation between the difference score in time estimates and post-task preference, *b* = 1.08, 95% CI = [1.02, 1.15], *z* = 2.54, *p* = .011, resulting in individuals being 1.08 times more likely to prefer the organisation-based strategy for every minute that it was perceived to be faster than the organisation-based strategy. The intercept did not reach significance when the relative perceived time difference was statistically controlled at 0, *b* = 2.06, 95% CI = [1.00, 4.50], *z* = 1.92, *p* = .055.

**Figure 4.**
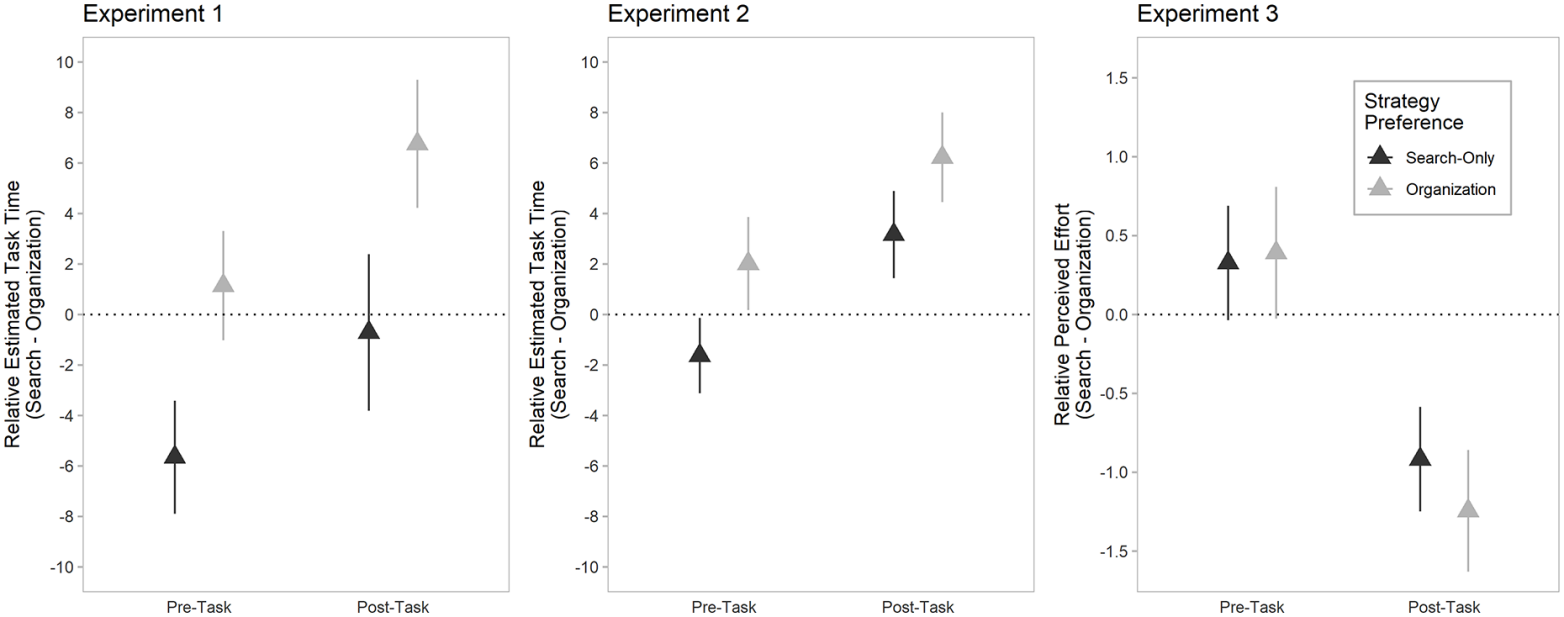
Mean difference score (estimates provided for the search-only strategy minus estimate provided for the organisation-based strategy) across experiments as a function of individuals’ strategy preference, both pre- and post-task. Error bars represent 95% confidence intervals. Note that in Experiments 1 and 2, difference scores are based on estimated, task while in Experiment 3, difference scores are based on perceived task effort.

In addition, we also examined the role of the actual task that individuals completed and whether it had any bearing on individuals’ post-task strategy preference. That is, it is possible that individuals’ experience with the search task itself influenced their downstream strategy preference. To do so, we added two additional factors to the logistic regression model containing post-task time estimation: actual time taken to complete the search task and the strategy that individuals were assigned to use. Aside from relative perceived task time, neither assigned strategy, *b* = 0.96, 95% CI = [0.44, 2.14], *z* = 0.09, *p* = .926, nor actual task time, *b* = 0.95, 95% CI = [0.85, 1.05], *z* = 0.87, *p* = .384, were significant predictors of post-task strategy selection. A likelihood ratio test revealed that the current model did not significantly improve the model fit compared with the original model (i.e., a model that contains only perceived time), χ^2^(2) = 0.83, *p* = .659. The model summary of participants’ pre- and post-task strategy preference can be found in Table 1, and the difference scores per group are depicted in Figure 4.

**Table 1.** Logistic regression model examining pre- and post-task strategy in Experiment 1, using relative time difference (time estimate for the search strategy minus time estimate for the organisation-based strategy) as the predictor.

Predictors	Pre-task strategy preference				Post-task strategy preference				Post-task strategy preference with additional predictors			
	Odds ratios	CI	Statistic	*p* value	Odds ratios	CI	Statistic	*p* value	Odds ratios	CI	Statistic	*p* value
Intercept	0.33	0.15–0.64	−3.05	<.001	2.06	1.00–4.50	1.92	.055	2.12	1.01–4.78	1.94	.053
Relative perceived task time	1.12	1.04–1.24	2.69	.007	1.08	1.02–1.15	2.54	.011	1.08	1.02–1.15	2.37	.018
Actual task time									0.95	0.85–1.05	−0.87	.384
Assigned strategy									0.96	0.44–2.14	−0.09	.926
Observations	53				41				41			
*R*^2^ Tjur	.171				.201				.218			

CI: confidence interval.

##### Strategy preference reversal pre- versus post-task

In addition to having separate analyses for individuals’ pre- and post-task strategy preference, we also examined the degree to which individuals’ strategy preferences changed before versus after performing the search task. Because the same individual provided multiple responses in the experiment (i.e., one set before and one set after completing the task), we applied logistic mixed-effects modelling to account for within-subjects variance. Relative time difference (i.e., time estimate for the search-only strategy minus that of the organise-first strategy) and task phase (pre- vs. post-task) were used in this model to predict individuals’ strategy preference. For this and similar analyses, we only allowed the intercept to vary per individual in the random effects structure, as there were too few data points for more complex models to reach convergence.^2^ A summary of the model specification and output can be found in Table 2.

**Table 2.** Logistic mixed-effects model examining strategy selection pre- compared with pos-task in Experiment 1, with task phase (pre- vs. post-task) and relative perceived task time as predictors.

Predictors	Pre- vs. post-task strategy preference			
	Odds ratios	CI	Statistic	*p* value
Intercept	0.72	0.42–1.25	−1.17	.241
Relative perceived task time	1.10	1.04–1.17	3.19	.001
Task phase	0.42	0.24–0.76	−2.87	.004
Random effects				
σ^2^	3.29			
τ_00 ID_	0.65			
ICC	0.16			
N _ID_	53			
Observations	106			
Marginal *R*^2^/Conditional *R*^2^	.406/.504			

ICC: intraclass correlation coefficient; CI: confidence interval.

Sum contrast coding was used for categorical variables.

Results indicated that relative time difference significantly predicted strategy preference, *b* = 1.10, 95% CI = [1.04, 1.17], *z* = 3.19, *p* = .001. Specifically, participants were 1.10 times more likely to prefer the organisation-based strategy for every minute it was perceived faster relative to the search-only strategy. Importantly, there was a significant shift in individuals’ strategy preference pre- versus post-task such that individuals were significantly less likely to prefer an organisation-based strategy pre- rather than post-task, *b* = 0.42, 95% CI = [0.24, 0.76], *z* = 2.78, *p* = .004.

### Discussion

In Experiment 1, participants overwhelmingly preferred a search-only strategy prior to engaging in the task, even though participants showed no difference in their actual task completion time across the two task strategies. Strategy preference was driven, in part, by participants’ estimated task time, with individuals preferring the strategy that they thought would take the least amount of time. However, perceived task time considerations did not completely explain the strategy preference, given that participants were still more likely to choose the search-only strategy even when the estimated task time did not differ (via statistically controlling for time) between the two strategies. Post-task, participants’ strategy preference reversed such that they preferred an organisation-based strategy. These preferences were again predicted by relative perceived task time, with individuals preferring the strategy that they perceived to be faster. Furthermore, in a follow-up analysis, we did not find evidence that actual strategy assignment or actual task completion time significantly predict strategy preference in this sample.

Given that relative perceived task time significantly predicted individuals’ strategy preferences both pre- and post-task, these results lend support to the time-minimisation hypothesis that individuals would choose the strategy that is expected to take less time. However, the observed strategy preferences were not fully explained by this factor, with individuals showing an initial bias against a search strategy that involved organisation, as indicated by the model intercept. These preferences are especially interesting, as participants’ performance using randomly assigned strategies showed that they were no faster at completing the search task regardless of which strategy was used. In Experiment 2, we attempt to replicate these results and further examine why these biases in strategy preference may have emerged.

## Experiment 2

In Experiment 1, we found that individuals preferred whichever strategy was thought to minimise perceived task time. However, the perceived time costs associated with each strategy could not—in their entirety—explain the observed preference for directly searching for the target pieces prior to engaging in the search task. The goal of the current experiment was to attempt to gain a better understanding of why individuals exhibited these kinds of systematic biases in their strategy preference, focusing on examining the systematic preference against organisation pre-task.

One possible reason for the reported pre-task preference to directly search for the target pieces may reflect a desire to progress continuously in the task space, as opposed to delaying progress on the primary task (i.e., search) until after a period of organisation. Although spatial organisation can allow certain objects or information to become more salient in the environment (Kirsh, 1995) and lead to accelerated progress in the primary task (e.g., search), the act of organisation in and of itself could be argued to not *directly* bring individuals closer to completing said task (e.g., finding items in the search task). For example, individuals who organise their closet systematically will be able to locate individual clothing items more easily when they go to search for them in the future. However, the act of organising one’s closet in and of itself does not lead to progress in the future search task but merely facilitates it. Put differently, by the time individuals have finished rearranging their task space, they are no closer to their goal of locating target items than they were before rearranging their task space (i.e., assuming they did not start on the search task while performing the organisation task). In the context of the current experiments, participants assigned to the organisation-based strategy were required to organise all the Lego pieces prior to engaging in search. As such, participants may anticipate a lack of progress at the start of the task. If this is the case, then the expectation of this perceived lack of progress may have contributed to the observed tendency for individuals to avoid choosing an organisation-based strategy pre-task. Thus, in Experiment 2, we assessed how individuals perceived their progress across the search- and organisation-based strategies to understand the systematic bias observed in individuals’ strategy preference pre-task. If participants’ perceived task progress reflected their objective progress on the primary search task, then we would expect individuals’ reported task progress to be relatively linear as a function of time for those assigned to the search-only strategy. However, we expect individuals assigned to complete the task using the organisation-based strategy to show a non-linear pattern in their self-reported task progress. Specifically, we would expect little to no perceived progress in the task during the initial organisation phase, and accelerated progress during the latter search phase.

### Methods

#### Participants

A total of 50 students (37 female) from the University of Waterloo were recruited to participate in our study for course credit. The mean age of this sample was 20.06 years (*SD* = 2.71); all participants reported normal or corrected-to-normal vision.

Prior to exclusion, 25 participants were assigned to each of the two strategies. After the exclusion criteria were applied, there were 22 participants assigned the search-only strategy and 20 for the organisation-based strategy.

#### Materials and procedure

In Experiment 2, participants were provided with voice prompts during the assigned search task using PsychoPy (Version 1.83.1 Peirce et al., 2019) on a Windows PC. The prompts directed participants to stop what they were doing and to orally respond, in minutes, how much time they thought was still needed to complete the task. After a 3-s response window, participants were given a separate prompt that asked them to resume their task. Prompts were delivered at pseudo-random time points within a 5-min interval, ensuring that participants would be provided with a prompt every 5 min on average, until they have completed the search task. As subjective progress of time was critical in the current experiment, participants were told to put away their cellphones and watches at the start of the experiment. With the exception of added voice prompts throughout the experiment, the procedure was identical to Experiment 1.

To mitigate technical issues, we encountered with the previous recording device, and to enhance video quality, we switched the recording device to a 1080HD Logitech C920.

### Results

As with Experiment 1, we removed eight participants’ objective search time as well as their responses during and after search from analyses due to equipment failure (2), improper task completion (4), or task incompletion (2). Unless otherwise noted, the pattern of results remained the same when these subjects were removed from analysis.

#### Objective task time

A Welch’s two-sample *t* test indicated that individuals assigned to the search-only strategy (*M* = 26.28, *SD* = 6.92) took just as long as those assigned to the organisation-based strategy, *M* = 26.91, *SD* = 6.38, *t*(39.99) = 0.31, *p* = .761, *g* = 0.09. On average, individuals spent 11.86 min (*SD* = 3.26) in the organisation phase.

#### Estimated task completion time

A paired-samples *t* test found no difference in overall time estimates provided for the search-only (*M* = 28.42, *SD* = 11.12) and organisation-based strategies, M = 27.78, *SD* = 11.50, *t*(49) = 0.53, *p* = .601, *g* = 0.06, prior to the search task. The average estimated completion time for the organisation phase was 11.16 min (*SD* = 5.54). However, post-task, individuals estimated that the search-only strategy (*M* = 31.52, *SD* = 10.00) would take significantly longer than the organisation-based strategy, *M* = 26.5, *SD* = 7.67, *t*(41) = 4.08, *p* < .001, *g* = 0.54. The post-task organisation phase was estimated to take 11.56 min (*SD* = 5.33).

**Figure 5.**
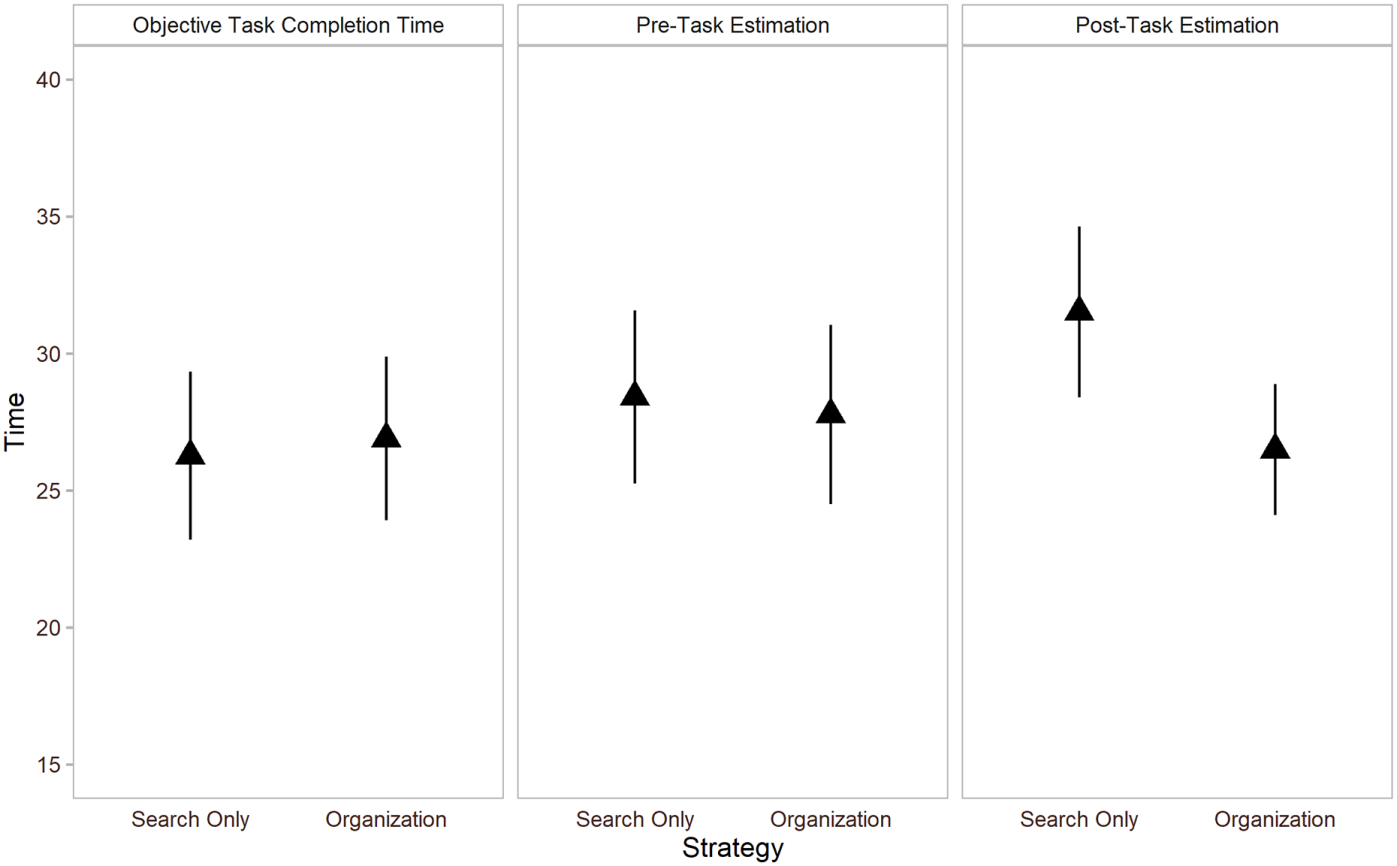
Objective task completion time as a function of assigned strategy (left) and estimated task time using either strategy (centre and right) in Experiment 2. Error bars represent 95% confidence intervals.

Of the 50 participants in our sample pre-task, there were 23 who perceived that an organisation-based strategy would result in faster task completion, 18 who perceived that the search-only strategy would be faster, and the remaining 9 who perceived that the two strategies take the same amount of time. Post-task, 28 of the 42 participants estimated that the organisation-based strategy would be faster, 7 perceived that the search-only strategy would result in faster task completion, while 7 estimated that they would take the same amount of time. Results for objective as well as estimated task completion time are shown in Figure 5.

#### Strategy preference

##### Pre-task strategy preference

Replicating the result of Experiment 1, the majority of individuals (70.0%) preferred the search-only compared with the organisation-based strategy, χ^2^(1, *N* = 50) = 8.00, *p* = .005. Similarly, a logistic regression indicated that the likelihood of selecting an organisation-based strategy increased by 1.22 times for every minute that the organisation-based strategy was perceived to be faster than the search-only strategy, *b* = 1.22, 95% CI = [1.09, 1.43], *z* = 3.05, *p* < .001. However, even when relative perceived task time was statistically held constant at 0, there was still an overwhelming preference for the search-only strategy as indicated by the significant intercept in the model such that it was 4 times more for individuals to prefer the search-only strategy over the organisation-based strategy, *b* = 0.25, 95% CI = [0.09, 055], *z* = 3.13, *p* < .001.

##### Post-task strategy preference

Post-task, 81.0% of individuals preferred an organisation-based strategy overall, χ^2^(1, *N* = 42) = 16.10, *p* < .001. Figure 3 provides a visual depiction of individuals’ pre- and post-task strategy preference. However, a logistic regression using relative estimated task time as a predictor did not significantly predict post-task preference, *b* = 1.07, 95% CI = [0.87, 1.19], *z* = 1.37, *p* = .172. The intercept remained significant, such that individuals were 3.29 times more likely to prefer an organisation-based strategy over a search-only strategy when relative perceived task time was statistically held constant at 0, *b* = 3.29, 95% CI = [1.48, 8.04], *z* = 2.81, *p* < .001.

Like in Experiment 1, we also examined whether the addition of actual task time and assigned task strategy predicted individuals’ post-task strategy preference in a separate model. Actual task time was not a significant predictor of post-task strategy preference, *b* = 1.06, 95% CI = [0.93, 1.24], *z* = 0.85, *p* = .398, nor was assigned task strategy, *b* = 0.34, 95% CI = [0.07, 0.91], *z* = 1.84, *p* = .066. A likelihood ratio test between the current model with additional predictors and the previous model that included only relative perceived task time failed to reach significance, χ^2^(2) = 5.94, *p* = .051. The model summary of participants’ pre- and post-task strategy preference can be found in Table 3. See Figure 4 for a visual depiction of the results.

**Table 3.** Logistic regression model examining pre- and post-task strategy in Experiment 2, using relative time difference (time estimate for the search strategy minus time estimate for the organisation-based strategy) as the predictor.

Predictors	Pre-task strategy preference				Post-task strategy preference				Post-task strategy preference with additional predictors			
	Odds ratios	CI	Statistic	*p* value	Odds ratios	CI	Statistic	*p* value	Odds ratios	CI	Statistic	*p* value
Intercept	0.25	0.09–0.55	−3.13	.002	3.29	1.48–8.04	2.81	.005	5.70	2.02–27.68	2.79	.005
Relative perceived task time	1.22	1.09–1.43	3.05	.002	1.07	0.97–1.19	1.37	.172	1.08	0.96–1.24	1.26	.208
Actual task time									1.06	0.93–1.24	0.85	.398
Assigned strategy									0.34	0.07–0.91	−1.84	.066
Observations	50				42				42			
*R*^2^ Tjur	.311				.040				.190			

CI: confidence interval.

##### Strategy preference reversal pre- versus post-task

As in Experiment 1, a logistic mixed-effects model was conducted to further compare individuals’ preference pre- versus post-task. Overall, individuals were 1.5 times more likely to prefer the organisation-based strategy for every minute that it was perceived to be faster relative to the search-only strategy, *b* = 1.50, 95% CI = [1.02, 2.21], *z* = 2.04, *p* = .041. Furthermore, individuals were also significantly less likely to prefer an organisation-based strategy pre-compared with post-task, *b* = 0.04, 95% CI = [0.00, 0.60], *z* = 2.34, *p* = .019. A summary of the model specification and outputs can be found in Table 4.

**Table 4.** Logistic mixed-effects model examining strategy selection pre- compared with post-task in Experiment 2, with task phase (pre- vs. post-task) and relative perceived task time as predictors.

Predictors	Pre- vs. post-task strategy preference			
	Odds ratios	CI	Statistic	*p* value
Intercept	0.75	0.12–4.63	−0.30	.761
Task phase	0.04	0.00–0.60	−2.34	.019
Time difference	1.50	1.02–2.21	2.04	.041
Random effects				
σ^2^	3.29			
τ_00 ID_	31.55			
ICC	0.91			
N _ID_	50			
Observations	98			
Marginal *R*^2^/Conditional *R*^2^	.452/.948			

ICC: intraclass correlation coefficient; CI: confidence interval.

Sum contrast coding was used for categorical variables.

#### Perceived task progress

Critical to the notion that individuals might perceive progress differently when using a search-only versus organisation-based strategy is that the former should exhibit a relatively linear relation between actual and self-reported task progress, whereas the latter is expected to be non-linear (i.e., that there would be little perceived task progress during the organisation phase of the task). Given that our critical dependent variable is task time remaining, we predict that a concave quadratic equation should better fit the data.

**Figure 6.**
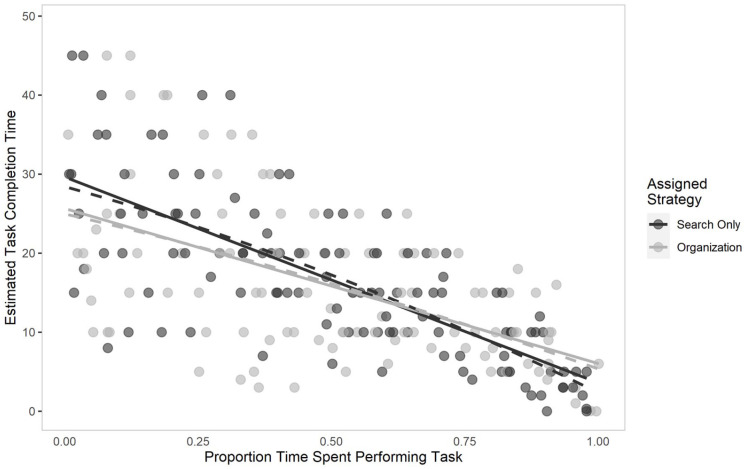
Estimated task completion time as a function of the proportion of time spent performing the Lego search task in Experiment 2. The darker data points represent participants’ responses in the assigned search-only strategy. Solid lines show the best linear fit for each assigned strategy, while dashed lines show the best curvilinear fit.

The data are depicted in Figure 6. To test this idea, we constructed a linear mixed-effects model using maximum likelihood estimation. To account for the fact that some participants completed the task more quickly than others, we divided the time that a given prompt was delivered to participants by the objective task completion time to derive a more standardised measure of time on task. As such, assigned task strategy (search-only vs. organisation-based), the first- and second-order polynomial term of proportion time on task, as well as the interaction term between the second-order polynomial and assigned strategy were included as predictors, and individuals’ estimated completion time was used as the criterion variable. As including a slope of proportion time in the random effects structure yielded a singular model, the model described below is the simplest model wherein only the intercept was allowed to vary by individual. Note that we do not report the estimate for the first-order polynomial term of standardised time on task below, as it represents the instantaneous rate of change when the x-intercept is at 0 (though see Table 3 for a full model summary). Estimated task completion time did not differ as a function of assigned strategy, *b* = 0.81, 95% CI = [−1.12, 2.74], *t* = 0.82, *p* = .410. The second-order polynomial term of proportion time on task was not a significant predictor, *b* = −2.59, 95% CI = [−12.55, 7.37], *t* = 0.51, *p* = .610, nor was there a significant interaction, *b* = −3.54, 95% CI = [−13.47, 6.39], *t* = 0.70, *p* = .484. In other words, we found no evidence for a curvilinear relation between proportion time spent on task and perceived task progress.

To examine the linear relation between proportion time and perceived task progress, we conducted a second set of linear mixed-effects models, with the second-order polynomial term removed. After model comparison, we allowed both the intercept and slope for proportion time to vary for each participant, as there was considerable variability across individuals in their estimates across proportion of time on task (σ^2^ = 182.64); this model also had a significantly better model fit than a model with just a random intercept structure, χ^2^(2) = 83.98, *p* < .001. Results showed that there was a significant linear relation between proportion time spent on task and estimated task time remaining, *b* = −21.86, 95% CI = [−26.35, −17.37], *t* = 9.55, *p* < .001, though overall estimates did not differ as a function of assigned strategy, *b* = 0.81, 95% CI = [−0.91, 2.52], *t* = 0.92, *p* = .359. The interaction term was not a significant predictor, *b* = −3.84, 95% CI = [−8.33, 0.65], *t* = 1.68, *p* = .093. The model summary for the linear term only model is also included in Table 5.

**Table 5.** Linear mixed-effects models examining whether perceived task progress in Experiment 2 differed between the two assigned strategies as a function of proportion time (linear model) or its squared term (curvilinear model).

Predictors	Best-fitting linear model				Best-fitting curvilinear model			
	Estimates	CI	Statistic	*p* value	Estimates	CI	Statistic	*p* value
Intercept	15.49	13.78 to 17.21	17.66	<.001	15.98	14.05 to 17.91	16.24	<.001
Proportion time	−21.86	−26.35 to −17.37	−9.55	<.001	−22.10	−24.73 to −19.48	−16.52	<.001
Assigned strategy	0.81	−0.91 to 2.52	0.92	.359	0.81	−1.12 to 2.74	0.82	.410
Proportion time × Assigned strategy	−3.84	−8.33 to 0.65	−1.68	.093				
Proportion time^2^					−2.59	−12.55 to 7.37	−0.51	.610
Proportion time^2^ × Assigned strategy					−3.54	−13.47 to 6.39	−0.70	.484
Random effects								
σ^2^	13.50				31.01			
τ_00_	29.34 _ID_				26.99 _ID_			
τ_11_	182.65 _ID.Time_							
ρ_01_	−0.73 _ID_							
ICC	0.77				0.47			
N	42 _ID_				42 _ID_			
Observations	220				220			
Marginal *R*^2^/Conditional *R*^2^	.422/.865				.411/.685			

ICC: intraclass correlation coefficient; CI: confidence interval.

Estimates are unstandardized *b* weights. Sum contrast coding was used for categorical variables.

### Discussion

In Experiment 2, we replicated the finding that prior to engaging in the task, individuals preferred strategies they thought would take less time overall. Again, individuals had an overall pre-task preference towards a search-only strategy. However, this preference could also not be fully explained by relative perceived task time estimates alone, given that individuals still preferred the search-only strategy when the relative perceived task time difference was at 0. We also replicated individuals’ strategy preference switch post-task such that individuals overwhelmingly preferred an organisation-based strategy. Although the odds ratio was comparable to Experiment 1, relative perceived task time did not significantly predict post-task strategy preference in the current experiment. Furthermore, neither actual task completion time nor assigned task strategy significantly predicted post-task strategy preference. We also did not find evidence supporting the idea that perceived task progress—as measured by participants’ estimated task time remaining—differed between assigned strategies. We address these results in detail below.

Although we find that relative perceived task time predicted individuals’ pre-task strategy preference across both experiments, this relation is less clear post-task, with relative perceived task time being a significant predictor in the model for post-task preference in Experiment 1 but a non-significant predictor in Experiment 2. Given that the odds ratios across the two experiments were comparable (i.e., 1.08 in Experiment 1 vs. 1.07 in Experiment 2) and that there was a reduction in the number of participants post-task versus pre-task (due to technical issues or task exclusion criteria), it is possible that a larger sample would yield an effect. To address this, we pooled data across the two experiments and conducted an exploratory logistic regression with experiment (Experiment 1 vs. 2) and relative perceived task time as well as their interaction term as predictors, and post-choice strategy preference as the criterion. With a combined sample (*N* = 83), relative perceived task time was a significant predictor of post-task strategy preference, with individuals being 1.07 times more likely to prefer the organisation-based strategy for every minute it was perceived to be faster than the search-only strategy, *b* = 1.07, 95% CI = [1.02, 1.14], *z* = 2.47, *p* = .014. Furthermore, the intercept in this model was also statistically significant, with individuals being 2.6 times more likely to prefer the organisation-based strategy relative to the search-only strategy across both experiments when relative perceived time difference was held constant at 0, *b* = 2.60, 95% CI = [1.52, 4.65], *z* = 3.37, *p* = .001. Experiment did not significantly predict strategy preference across the two experiments, *b* = 0.79, 95% CI = [0.45, 1.38], *z* = 0.82, *p* = .411, nor did it interact with relative perceived task time, *b* = 1.00, 95% CI = [0.95, 1.06], *z* = 0.11, *p* = .915. A summary of these results is provided in Table 6. Based on the pooled data, it appears that relative perceived task time—both pre- and post-task—predicts individuals’ chosen strategies.

**Table 6.** Logistic regression model examining post-task strategy pooling data from Experiments 1 and 2, using relative time difference (time estimate for the search strategy minus time estimate for the organisation-based strategy) as the predictor.

Predictors	Post-task strategy preference			
	Odds ratios	CI	Statistic	*p* value
Intercept	2.60	1.52–4.65	3.37	.001
Experiment	0.79	0.45–1.38	−0.82	.411
Time difference	1.07	1.02–1.14	2.47	.014
Experiment × Time difference	1.00	0.95–1.06	0.11	.915
Observations	83			
*R*^2^ Tjur	.152			

CI: confidence interval.

Sum contrast coding was used for categorical variables. Note that the experiment was not a significant moderator.

Contrary to our prediction that individuals perceived task progress differently when completing the task using the search-only versus organisation-based strategy, individuals assigned to the organisation condition did not show a non-linear pattern in their reported time estimations. In fact, follow-up analyses indicated that individuals in both conditions showed a linear relation in their estimates over time. One possible explanation for the lack of difference in perceived task progress across the two strategies is that our measure of progress (i.e., time) may not have captured the subjective experience of task progress accurately. Because individuals were asked to provide the amount of time they thought was remaining on the task—an indirect measure of perceived task progress—this may have inadvertently caused them to focus on how much time they have already spent on the entire task, rather than reflecting on the amount of progress they have made. In light of this possibility, we examine the perception of task progress across the different strategies in the next experiment by employing a more direct measure of perceived task progress.

## Experiment 3

In both experiments described thus far, we find that individuals attempt to minimise perceived task time when selecting whether to organise or not in a search task. Perceived time, however, only *partially* explained individuals’ strategy preference pre- and post-task; even accounting for perceived task time, individuals were still biased towards one preference or another. Notably, these results suggest that individuals’ decisions to engage in spatial organisation may be influenced by factors other than time considerations. One interesting candidate in this respect is perceived task effort. There is extensive research on the role of effort in strategy selection showing that individuals tend to avoid choosing task strategies or options that result in greater effort, whether perceived or as indexed by other proxies (Dunn et al., 2019; Dunn & Risko, 2019; Gilbert et al., 2020; Kool et al., 2010; Shenhav et al., 2017; Westbrook et al., 2013; Zipf, 2012). While some researchers have argued that individuals’ tendency to minimise task time reflects an effort minimisation strategy (Gray & Fu, 2004; Gray et al., 2006), there is evidence to suggest that effort-based decisions need not be based on time costs associated with a task (Dunn et al., 2019; Dunn & Risko, 2016, 2019; Kool et al., 2010; Westbrook et al., 2013). Indeed, individuals might be willing to endure additional time costs to avoid cognitively effortful lines of action (e.g., Kool et al., 2010). Like time, the relation between effort and strategy choice is likely a complex one. For example, Inzlicht and colleagues (2018) discussed the curious tension between the seemingly well-accepted notion of humans as cognitive-misers or effort minimisers with the observation that at least some individuals (in some contexts) appeared to seek out effortful activities. Nevertheless, what is clear from the literature is that effort represents an additional consideration (either as a cost or benefit) separate from perceived task time, which may drive individuals’ decision to select a given search strategy. As such, we examined whether individuals’ preferences for organisation-based strategies were related to the perceived effort of the associated strategy.

In addition to examining whether perceived task effort would influence search strategy selection, the current experiment also attempted to re-examine individuals’ perception of task progress across the two strategies using a different approach. In the current experiment, we asked participants to indicate how much progress they think they have made on the task using a progress bar, rather than asking participants to provide verbal estimates of how much time they anticipate needing to complete the remainder of the task. Similar to our prediction in Experiment 2, individuals assigned to use the search-only strategy should perceive a relatively linear relation between perceived progress and time spent on task; however, individuals who use the organisation-based strategy should experience little to no progress during the organisation phase, but perceived task progress should increase at an accelerated rate during the search phase of the task.

### Methods

#### Participants

A total of 49 students (43 female; 1 unknown) from the University of Waterloo were recruited to participate in our study for course credit. The ages of two individuals were not reported. For the remaining sample, the mean age was 19.49 years (*SD* = 2.08); all participants reported normal or corrected-to-normal vision.

Prior to exclusion, 26 participants were assigned the search-only strategy, while 23 were assigned the organisation-based strategy. After the exclusion criteria were applied, the number of participants was 25 and 21 in the respective conditions.

#### Materials and procedure

The overall task procedure was the same as in Experiments 1 and 2. However, rather than asking participants to estimate how many minutes it would take to complete the task using each strategy, participants were asked to rate how effortful each strategy seemed on a 6-point Likert-type scale ranging from 1 to 6.

Participants in Experiment 3 were also given voice prompts in the same manner as in Experiment 2. These prompts directed participants to pause in their task and to draw a vertical line on a progress bar (printed on a letter-sized piece of paper provided to the participant by the experimenter) to indicate how much progress they think they have made in the task. As all the progress bars were printed on a single sheet of paper, participants could reference any of their previous progress markings. Participants in this study were also told to put away their cellphones and watches at the start of the experiment.

### Results

Three participants’ objective search time and their responses during and after search were removed from analyses due to equipment failure (1), improper task completion (1), or task incompletion (1). Unless indicated otherwise, the pattern of results remained the same when these subjects were removed from the analyses.

#### Objective task time

In examining objective task performance, a Welch’s two-sample *t* test confirmed that individuals assigned to the organisation-based strategy (*M* = 29.44, *SD* = 5.00) took just as long as those assigned to the search-only strategy (*M* = 29.84, *SD* = 9.11), *t*(38.37) = 0.18, *p* = .855, *g* = 0.05). On average, individuals using the organisation-based strategy spent 13.68 min (*SD* = 3.24) organising the Lego pieces.

#### Estimated task effort

We found no difference in perceived task effort across the organisation-based (*M* = 3.49, *SD* = 1.31) and search-only strategy, *M* = 3.90, *SD* = 1.10, *t*(48) = 1.48, *p* = .146, *g* = 0.33, prior to the search task using a paired-sample *t* test. Interestingly, individuals rated the organisation-based strategy as being more effortful (*M* = 4.39, *SD* = 1.44) than the search-only strategy post-task, *M* = 3.32, *SD* = 1.62, *t*(45) = 4.06, *p* < .001, *g* = 0.74.

**Figure 7.**
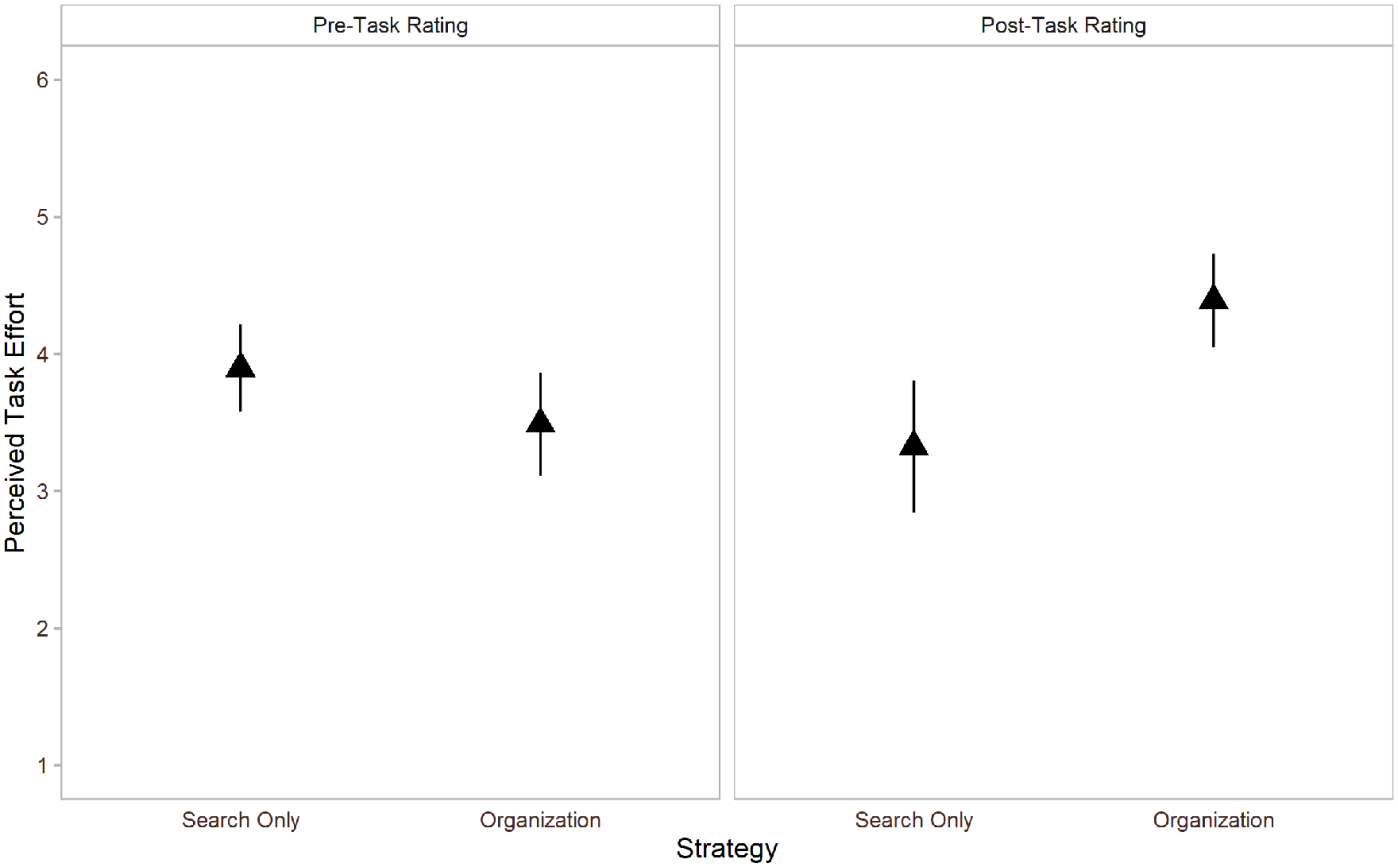
Self-reported task effort ratings on a 6-point Likert-type scale in Experiment 3. Error bars represent 95% confidence intervals.

Pre-task, 26 of the 49 participants rated the organisation-based strategy as being easier, 18 rated the search-only strategy to be easier, and 5 rated them as being equally effortful. Post-task, 12 of the 46 participants rated the organisation-based strategy as being easier, 31 rated the search-only strategy as being easier, and 3 rated them as being equally effortful. Results for objective as well as estimated task completion time are shown in Figure 7.

#### Strategy preference

##### Pre-task strategy preference

Prior to the search task, 73.5% of individuals preferred the search-only strategy, compared with an organisation-based strategy, χ^2^(1, *N* = 49) = 10.80, *p* = .001. Similar to Experiments 1 and 2, a relative effort score was calculated by subtracting individuals’ effort ratings for the organisation-based strategy from the ratings of the search-only strategy. Again, positive values indicate that individuals perceived the organisation-based strategy as being less effortful. A logistic regression was conducted using the difference score in perceived effort to predict individuals’ strategy preference. We found that effort ratings pre-task did not influence which strategy individuals chose, *b* = 1.11, 95% CI = [0.80, 1.60], *z* = 0.62, *p* = .534; the intercept in the model remained significant, such that individuals were 2.94 times more likely to prefer a search-only strategy over an organisation-based strategy when relative task effort was statistically controlled for at 0, *b* = 0.34, 95% CI = [0.17, 0.64], *z* = 3.15, *p* = .002.

##### Post-task strategy preference

Like in previous experiments, most individuals (71.7%) preferred an organisation-based strategy post-task, χ^2^(1, *N* = 46) = 8.70, *p* = .003. Figure 3 provides a visual depiction of individuals’ pre- and post-task strategy preference. When relative post-task effort was used as a predictor of strategy preference, it was not significant, *b* = 0.73, 95% CI = [0.48, 1.06], *z* = 1.60, *p* = .109. When relative task effort was held at 0, the intercept in the model was not statistically significant, *b* = 1.94, 95% CI = [0.96, 4.05], *z* = 1.82, *p* = .069. Note that while individuals preferred an organisation-based strategy at the aggregate level, the model intercept represents only individuals’ preference when task effort was statistically held constant at 0, rather than the group mean.

When assigned task strategy and actual task time were added as predictors in a separate model, neither actual task time, *b* = 1.09, 95% CI = [0.98, 1.23], *z* = 1.46, *p* = .145, nor assigned task strategy, *b* = 0.59, 95% CI = [0.26, 1.21], *z* = 1.39, *p* = .164, were significant predictors of post-task strategy preference. A likelihood ratio test revealed that the current model was not statistically better than the original model that included only relative perceived task effort, χ^2^(2) = 4.86, *p* = .088. The model summaries for individuals’ pre- and post-strategy preferences are shown in Table 7, and a visual depiction of the results can be found in Figure 4.

**Table 7.** Logistic regression model examining pre- and post-task strategy in Experiment 3, using relative perceived effort (perceived effort for the search strategy minus time estimate for the organisation-based strategy) as the predictor.

Predictors	Pre-task strategy preference				Post-task strategy preference				Post-task strategy preference with additional predictors			
	Odds ratios	CI	Statistic	*p* value	Odds ratios	CI	Statistic	*p* value	Odds ratios	CI	Statistic	*p* value
Intercept	0.34	0.17–0.64	−3.15	.002	1.94	0.96–4.05	1.82	.069	2.09	0.99–4.74	1.88	.06
Relative perceived task time	1.11	0.80–1.60	0.62	.534	0.73	0.48–1.06	−1.6	.109	0.74	0.46–1.11	−1.41	.158
Actual task time									1.09	0.98–1.23	1.46	.145
Assigned strategy									0.59	0.26–1.21	−1.39	.164
Observations	49				46				46			
*R*^2^ Tjur	.009				.053				.167			

CI: confidence interval.

##### Strategy preference reversal pre- versus post-task

A logistic mixed-effects model was conducted using perceived effort difference and task phase as predictors. Perceived effort did not significantly predict individuals’ strategy preference overall, *b* = 0.90, 95% CI = [0.66, 1.23], *z* = 0.67, *p* = .504. However, as with the previous two experiments, individuals were significantly less likely to prefer an organisation-based strategy pre- compared with post-task, *b* = 0.26, 95% CI = [0.10, 0.64], *z* = 2.91, *p* = .004. A summary of the model specification and outputs can be found in Table 8.

**Table 8.** Logistic mixed-effects model examining strategy selection pre- compared to pos-task In Experiment 3, with task phase (pre- vs. post-task) and relative perceived effort as predictors.

Predictors	Pre- vs. Post-Task Strategy Preference			
	Odds ratios	CI	Statistic	*p* value
Intercept	0.94	0.48–1.84	−0.19	.851
Task phase	0.26	0.10–0.64	−2.91	.004
Relative perceived effort	0.90	0.66–1.23	−0.67	.504
Random effects				
σ^2^	3.29			
τ_00 ID_	2.09			
ICC	0.39			
N _ID_	49			
Observations	96			
Marginal *R*^2^/Conditional *R*^2^	.283/.561			

ICC: intraclass correlation coefficient; CI: confidence interval.

Sum contrast coding was used for categorical variables.

#### Perceived progress

**Figure 8.**
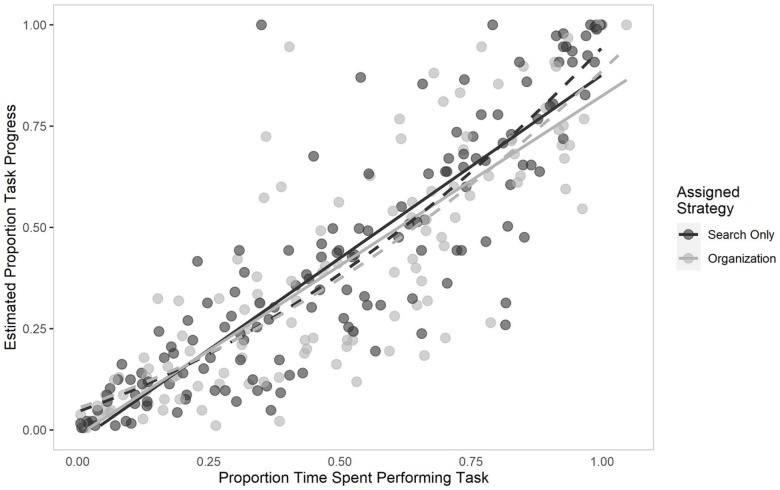
Perceived proportion task progress as a function of the proportion of time spent performing the Lego search task in Experiment 3. The darker data points represent participants’ responses in the assigned search-only strategy. Solid lines show the best linear fit for each assigned strategy, while dashed lines show the best curvilinear fit.

For Experiment 3, self-reported task progress was derived by measuring the distance between left end of the progress bar to the centre of the vertical line where individuals indicated how much progress they perceived to have made, and dividing that against the total length of the progress bar, resulting in a proportion score. A visual depiction of these results is shown in Figure 8. A linear mixed-effects model was constructed that used maximum likelihood estimation. Assigned strategy (search-only vs. organisation-based), the first- and second-order polynomial term of proportion time on task, as well as the interaction term between the second-order polynomial and assigned strategy were included as predictors, and individuals’ self-reported proportion progress was used as the criterion variable. As a more complex model with slopes varying for proportion time per individual resulted in a singular model, we opted for the simplest model in which only the intercept was allowed to vary per individual. Assigned strategy was not a significant predictor of proportion progress reported, *b* = 0.00, 95% CI = [−0.03, 0.04], *t* = 0.18, *p* = .858. The second-order polynomial term of proportion time on task was a significant predictor, *b* = 0.44, 95% CI = [0.28, 0.61], *t* = 5.21, *p* < .001, but this relation was not significantly moderated by assigned condition, *b* = 0.01, 95% CI = [−0.16, 0.17], *t* = 0.06, *p* = .953.

As with Experiment 2, we also constructed models with only linear terms, as well as their interaction, for comparison. After comparison, we chose a model wherein the slope of proportion time spent on task was allowed to vary per individual, as it had a significantly better model fit, χ^2^(2) = 9.75, *p* = .007. We found that there was a positive linear relation between proportion time spent on task and reported task progress, *b* = 0.86, 95% CI = [0.81, 0.91], *t* = 33.02, *p* < .001. Assigned strategy was not a significant predictor, *b* = 0.01, 95% CI = [−0.03, 0.04], *t* = 0.32, *p* = .750, nor was it moderated by time spent on task, *b* = 0.03, 95% CI = [−0.03, 0.08], *t* = 1.00, *p* = .319. The statistical model summary for both the linear and curvilinear models can be found in Table 9.

**Table 9.** Linear mixed-effects models examining whether perceived task progress in Experiment 3 differed between the two assigned strategies as a function of proportion time (linear model) or its squared term (curvilinear model).

Predictors	Linear model				Curvilinear model			
	Estimates	CI	Statistic	*p* value	Estimates	CI	Statistic	*p* value
Intercept	0.41	0.38 to 0.45	25.28	<.001	0.38	0.34 to 0.41	20.89	<.001
Proportion time	0.86	0.81 to 0.91	33.02	<.001	0.87	0.83 to 0.91	38.98	<.001
Assigned strategy	0.01	−0.03 to 0.04	0.32	.750	0.00	−0.03 to 0.04	0.18	.858
Proportion time × Assigned strategy	0.03	−0.03 to 0.08	1.00	.319				
Proportion time^2^					0.44	0.28 to 0.61	5.21	<.001
Proportion time^2^ × Assigned strategy					0.01	−0.16 to 0.17	0.06	.953
Random effects								
σ^2^	0.01				0.01			
τ_00_	0.01 _ID_				0.01 _ID_			
τ_11_	0.01 _ID.Progress.c_							
ρ_01_	1.00 _ID_							
ICC	0.46				0.47			
N	46 _ID_				46 _ID_			
Observations	275				275			
Marginal *R*^2^/Conditional *R*^2^	.740/.860				.754/.869			

ICC: intraclass correlation coefficient; CI: confidence interval.

Estimates are unstandardized *b* weights. Sum contrast coding was used for categorical variables.

### Discussion

The results in Experiment 3 replicated individuals’ strategy preferences in Experiments 1 and 2, in that individuals initially preferred a search-only strategy and switched to prefer an organisation-based strategy post-task. Interestingly, we did not find strong evidence that these preferences were explained by relative differences in self-reported task effort to support an effort minimisation account. First, there was no overall difference in perceived effort across the two strategies pre-task but nevertheless a strong preference for the search-only strategy. Post-task, individuals preferred an organisation-based strategy, a strategy that was rated as being overall more effortful at the aggregate level. Interestingly, despite these aggregate-level differences, relative perceived effort at the individual level did not predict individuals’ strategy preference either pre-or post-task. As noted earlier, the relation between effort and strategy choice is likely complex, and the notion that some individuals might seek out effortful activities (e.g., Inzlicht et al., 2018) seems worth considering in the present context. For example, organisation might reflect a kind of effort some individuals might enjoy (i.e., imposing order on a messy environment). Another worthwhile consideration is that the two strategies here might differ in terms of physical and cognitive effort; i.e., it is possible that one form of effort is in fact an important consideration for whether individuals would prefer one strategy over another here, whereas the other form of effort does not. Future work more closely examining the perception of effort associated with organisational strategies would be valuable.

In addition, we also re-examined individuals’ self-reported progress. Unlike in Experiment 2, where we observed a linear relation between self-reported task progress and time on task, we found that participant’s perceived task progress across both assigned strategies followed a non-linear trend such that the rate of task completion accelerated near the end of the task. Although we did not expect to find this pattern, the acceleration in perceived task progress near the end of the task could be attributed to an increasingly visible decrease in the number of Lego pieces in the task space as more target items are found. The fact that we did not find this non-linear pattern in Experiment 2 may speak to the fact that the two measures of task progress may indeed be tapping into slightly different interpretations of task progress. However, it is important to note that we also found strong evidence of a linear pattern across both Experiment 2 and 3, such that perceived task progress increased the more time individuals spent on the task, suggesting that the perception of task progress is overall relatively linear. Crucially, we found no difference in the form of the relation between perceived and actual task progress across the two different strategies. These results suggest that individuals do not differ in how they perceive task progress across an organisation-based versus search-only strategy. Thus, our idea that individuals’ perception of task progress caused the differences in preference for the two strategies pre-task was not supported.

## Combined analysis

Across three experiments, we observed a consistent pattern wherein individuals switched their preference post-task from a search-only to an organisation-based strategy. As we did not have any a priori predictions regarding this observation, we conducted exploratory analyses to examine why this may have been the case. Based on previous work (Hoeffler et al., 2006; Zhu & Risko, 2016), an individual’s past experiences and decisions have been reported to influence their future decisions and preferences. While we have attempted to do this by examining whether assigned strategy and actual task time were significant predictors of post-task strategy preference in each of the three experiments separately, it is possible that we were underpowered to be able to detect an effect. For example, though assigned strategy was not a significant predictor in each experiment separately, the estimates showed that the effects were all in the same direction (0.96 in Experiment 1; 0.34 in Experiment 2; 0.59 in Experiment 3). As such, we combined data across all three experiments to maximise power. In addition to examining whether individuals’ assigned search strategy and actual task completion time influenced their post-task strategy preference, we also added initial pre-task strategy preference as a predictor in the combined exploratory analysis.

**Figure 9.**
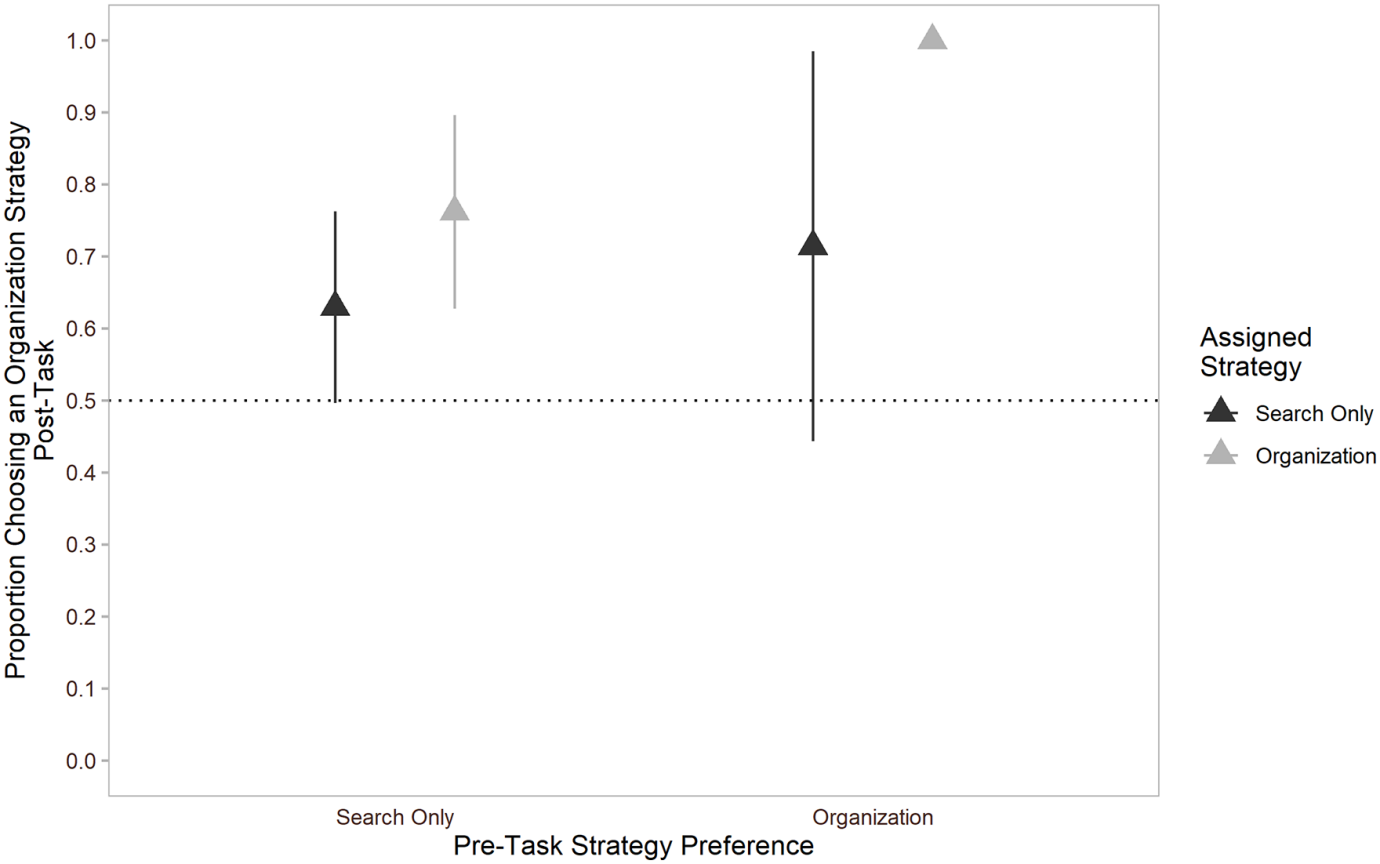
Mean proportion of individuals who preferred an organisation-based strategy post-task as a function of pre-task strategy preference and assigned search task across all experiments. Error bars represent 95% confidence intervals. Note that all individuals who both chose an organisation initially and assigned to this same strategy (*N* = 19) picked an organisation-based strategy post-task.

To do so, we conducted a logistic regression using assigned search strategy, actual task time, and pre-task preference as predictors, and individual’s post-task strategy as the criterion variable. We found that actual task time was not a significant predictor of post-task strategy preference, *b* = 1.02, 95% CI = [0.96, 1.08], *z* = 0.57, *p* = .567. Assigned strategy was a significant predictor of post-task preference; individuals assigned to the search-only strategy were less likely to choose an organisation strategy post-task, *b* = 0.62, 95% CI = [0.40, 0.94], *z* = 2.21, *p* = .027. Specifically, individuals who were assigned the search-only strategy were 1.62 times more likely to prefer the search-only strategy relative to the intercept, which represents the grand mean. That is, there was a tendency to stick with one’s assigned strategy. Pre-task preference, however, was not a significant predictor, *b* = 0.58, 95% CI = [0.30, 0.98], *z* = 1.85, *p* = .065.^3^ Overall, these results lend support to the idea that some aspects of an individual’s past history with that task can indeed influence their future strategy preference. It is important to note that the intercept in the current model, which reflects the grand mean, is also significant, *b* = 4.24, 95% CI = [2.48, 8.19], *z* = 4.84, *p* < .001, suggesting that the systematic bias towards organisation remained even when controlling for prior preference and task history. A summary of the logistic regression model results can be found in Table 10; Figure 9 depicts the results, split by pre-task preference and assigned task strategy.

**Table 10.** Logistic regression model examining post-task strategy pooling data across all three experiments, using actual task time, assigned task strategy, and pre-task strategy preference as predictors.

Predictors	Post-task strategy preference			
	Odds ratios	CI	Statistic	*p* value
Intercept	4.24	2.48–8.19	4.84	<.001
Actual task time	1.02	0.96–1.08	0.57	.567
Assigned strategy	0.62	0.40–0.94	−2.21	.027
Pre-task preference	0.58	0.30–0.99	−1.85	.065
Observations	129			
*R*^2^ Tjur	.074			

CI: confidence interval.

A model with an interaction term between pre-task preference and assigned strategy was attempted, but the model did not converge.

## General discussion

We set out to examine the degree to which individuals preferred to engage in spatial organisation before performing a primary task and tested a number of potential factors that may influence their decisions to do so. Overall, we identified a consistent pattern such that the majority of individuals avoided choosing an organisation-based strategy when asked about their preference between an organisation- or non-organisation-based strategy prior to engaging in a search task. Also consistent was the fact that we observed a reversal in strategy preference post-task such that most individuals preferred an organisation-based strategy. These results suggest that individuals had fairly systematic preferences prior to and after completing the search task. When we examined potential factors that could affect individuals’ preferences—namely, perceived time costs in Experiments 1 and 2, and task effort in Experiment 3—we found that the former was able to partially explain individuals’ preferred search strategies while there was little evidence to indicate that the latter did so.

The results presented in our studies provided evidence that perceived task time can play an important role when determining whether to decide to use a spatial organisation-based strategy. This is consistent with previous research (Gray & Boehm-Davis, 2000; Gray et al., 2006). Nonetheless, it is important to note that in the context of the current paradigm, we did not explicitly emphasise performance-oriented goals. Rather, participants were asked to complete the task at hand and, as such, it seems reasonable for participants to prefer whichever strategy that would lead them to minimise the amount of time devoted to it. It would be interesting to examine how individuals’ strategy preference might change if, e.g., they were rewarded for speed or accuracy (e.g., Weis & Wiese, 2019). While findings from the current experiments support the notion that individuals’ strategy preference reflects a consideration of perceived time, these results also demonstrate that perceived time costs were not the only consideration, as it could not wholly account for their preferences when we statically controlled for differences in perceived task time across strategies.

Although the current experiments cannot offer definitive explanations for why individuals showed systematic pre-task strategy preferences, Experiments 2 and 3 revealed that perceived task progress likely does not underlie the observed bias. As mentioned previously, when individuals engage in spatial organisation, they would—at least in one meaningful sense—not be making any progress on the primary task; however, individuals who engage in a search-only strategy would progress in the search task in a more linear manner. However, individuals do not appear to perceive task progress differently as a function of task strategy. The fact that task progress is perceived relatively linearly as a function of time on task suggests that individuals may perceive organisation as more than just an auxiliary action that helps to support a given primary task (e.g., search). In fact, rather than feeling a stall in task progress during an initial organisation phase that later accelerates during search, individuals who were assigned to use an organisation-based strategy perceived that task progress was made at the same rate regardless of whether they were engaging in the organisation or search phase of the task. As such, the current results suggest that individuals perceive that engaging in spatial organisation is still propelling them closer to the end goal—despite a lack of *visible* search progress in the number of target pieces found. Given the relatively small sample size, it is possible that we were underpowered in detecting the proposed moderation. However, it is important to also point out that we were exploring perceived progress as a potential explanation for the pre-task preference pattern we observed across each of the reported experiments. This effect (i.e., pre-task preference for search) was relatively large and robust, and our results have demonstrated that the samples in each experiment separately have sufficient power in detecting individuals’ systematic preference against organisation pre-task. As such, even if the samples in the current experiments failed to detect a small interaction with regard to perceived progress, it seems clear that the effect of assigned strategy on perceived progress is unlikely to provide a compelling explanation of the pre-task pattern, which was our goal for examining perceived progress.

The shift in individuals’ strategy preference pre- to post-task however could, to a degree, be explained by some aspects of an individual’s overall experience with the task. Specifically, individuals were more likely to choose a strategy that they were previously assigned to use to complete the search task. These results align with past findings that indicate that an individual’s past history with a task can significantly influence their future spatial decisions (Zhu & Risko, 2016). Intriguingly, regardless of one’s past strategy preferences or assigned strategies, individuals were still more likely to prefer an organisation-based strategy post-task. This suggests that having *any* experience with the task—regardless of one’s initial preference or assigned strategy—may shift individuals’ preferences from one that involves no organisation to an organisation-based strategy. However, it is also possible that individuals’ intention to engage in spatial organisation post-task would not translate into future action, as we did not ask participants to perform the search task again. That is, their stated desire to first organise a space prior to search was expressed under different conditions than their initial (pre-task) preference (i.e., when they knew they would have to actually perform the task). As there is extensive research on the misalignment between individuals’ intentions and actual behaviour (Auger & Devinney, 2007; Norberg et al., 2007; Polites et al., 2018), more work would be required to assess the degree to which the preferences reported post-task reflect individuals’ strategy preference if they knew they would have to perform the task.

In summary, the current experiments present preliminary evidence that individuals engage in cost-benefit-like judgements when deciding whether they preferred a spatial organisation-based strategies in the context of an everyday search task. While organising one’s space has been demonstrated to help reduce the amount of physical and cognitive demands associated with subsequent task performance (Berry et al., 2019; Kirsh, 1995; Solman & Kingstone, 2017a; Zhu & Risko, 2016), our results show that individuals also weigh the costs of doing so. In addition, we also show that an individual’s preference for engaging in spatial organisation may also depend on some aspects of their past experience or history with a given task. As such, the studies presented in this article shed light on a more nuanced perspective of whether—and the degree to which—individuals may choose to engage in spatial organisation, and provide an important first step for understanding the kinds of factors that may contribute towards such decisions.

## Notes

1. When we removed data from the same 12 subjects who were removed from post-task analyses, difference approached but did not reach significance between the estimates, with the search-only strategy being rated as being slightly faster (*M* = 28.78, *SD* = 14.53) than the organisation-based strategy—*M* = 32.12, *SD* = 19.05, *t*(40) = 1.81, *p* = .077, *g* = 0.18.

2. We attempted to include a model that included an interaction term between task phase and time, but due to model over-specification, we could not include this term in the model.

3. We attempted a model with an interaction term between pre-task preference and assigned strategy, but the interaction term could not be accurately estimated due to the limited number of observations in some of the conditions.
